# Does Liberal Prehospital and In-Hospital Tranexamic Acid Influence Outcome in Severely Injured Patients? A Prospective Cohort Study

**DOI:** 10.1007/s00268-021-06143-y

**Published:** 2021-04-29

**Authors:** Karlijn J. P. van Wessem, Luke P. H. Leenen

**Affiliations:** Department of Trauma Surgery, University Medical Center Utrecht, Heidelberglaan 100, 3584 CX Utrecht, The Netherlands

## Abstract

**Background:**

Early hemorrhage control is important in trauma-related death prevention. Tranexamic acid (TXA) has shown to be beneficial in patients in hemorrhagic shock, although widespread adoption might result in incorrect TXA administration leading to increased morbidity and mortality.

**Methods:**

A 7-year prospective cohort study with consecutive trauma patients admitted to a Level-1 Trauma Center ICU was performed to investigate administration of both pre- and in-hospital TXA and its relation to morbidity and mortality. Indication for prehospital and in-hospital TXA administration was (suspicion of) hemorrhagic shock, and/or systolic blood pressure (SBP) ≤ 90 mmHg. Demographics, data on physiology, resuscitation and outcomes were prospectively collected.

**Results:**

Four hundred and twenty-two patients (71% males, median ISS 29, 95% blunt injuries) were included. Even though TXA patients were more severely injured with more deranged physiology, no differences in outcome were noted. Overall, thrombo-embolic complication rate was 8%. In half the patients, hemorrhagic shock was the indication for prehospital TXA, whereas 79% of in-hospital TXA was given based on suspicion of hemorrhagic shock. Thirteen percent of patients with SBP ≤ 90 mmHg in ED received no TXA at all. Based on SBP alone, 22% of prehospital TXA and 25% of in-hospital TXA were justified.

**Conclusions:**

Despite being more severely injured, TXA patients had similar outcome compared to patients without TXA. Thrombo-embolic complication rate was low despite liberal use of both prehospital and in-hospital TXA. Caution should be exercised in selecting patients for TXA, although this might be challenging based on SBP alone in patients who do not yet show signs of deranged physiology on arrival in ED.

**Supplementary Information:**

The online version contains supplementary material available at 10.1007/s00268-021-06143-y.

## Background

Early hemorrhage control and adequate blood product transfusion are important in trauma-related death prevention [1, 2]. Hemostatic resuscitation prevents ongoing blood loss, restores volume status and corrects coagulopathy development [3]. Tranexamic acid (TXA) is an anti-fibrinolytic agent that acts by inhibiting plasminogen activation and fibrinolysis and promotes the ability to sustain formed clots [4]. The Clinical Randomization of an Anti-fibrinolytic in Significant Hemorrhage 2 (CRASH-2) trial showed statistically significant improvement in the rates of both overall mortality and in hemorrhage-caused mortality as a result of early administration of TXA in adults who sustained an injury within 8 h and had either significant hemorrhage, hypotension or who were considered to be at risk of significant hemorrhage [5]. These results have led to widespread incorporation of TXA in damage control resuscitation with low thresholds to administer TXA, including in prehospital settings. However, concerns have been raised that indiscriminate widespread adoption might result in TXA administration in the wrong patients, leading to increased morbidity and mortality [6–9]. Data in the literature have been contradicting, however, with others reporting no significant differences or even decreased adverse effects [10–14]. At present, it remains unclear what the exact mechanism behind TXA is and how it has reduced mortality in CRASH-2 trial, since there was no reduction in packed red blood cells (PRBC) transfusion between patients who received TXA and the ones who did not [5]. Data are still lacking regarding which trauma patients might benefit most, optimal dosing and timing and potential complications in both prehospital and in-hospital setting [4, 15].

Since most studies only focused on either prehospital or in-hospital TXA administration, we conducted a prospective population-based cohort study in polytrauma patients to investigate the indication of both pre- and in-hospital TXA administration and its relation to morbidity and mortality. We hypothesized that neither prehospital nor in-hospital TXA administration was related to increased morbidity or mortality.

## Materials and methods

A 7-year prospective population-based cohort study (starting November 2013) was undertaken to investigate outcomes in severely injured patients admitted to the Intensive Care Unit (ICU) of a major (Level-1) trauma center (University Medical Center Utrecht, The Netherlands). Details of the hospital and catchment area were previously described [16]. All consecutive polytrauma patients who were admitted to the adult ICU were included. ICU admission could be either directly from the emergency department (ED) or postoperatively after urgent surgery. Patients with isolated traumatic brain injury (TBI), asphyxiation, drowning and burns were excluded, because of potential different physiologic response to severe trauma and a significantly different mortality and morbidity profile [17, 18]. Isolated injury to the brain was defined as Abbreviated Injury Score (AIS) head ≥ 3 and AIS ≤ 2 in other regions.

All data were prospectively collected by both authors and included demographics, shock and resuscitation parameters. Administration of both crystalloid and blood products including packed red blood cells (PRBC), fresh frozen plasma (FFP) and platelets (PLT) was documented in the first 24 h after admission. Additionally, prehospital and in-hospital administration (in ED, OR, ≤ 8 h and ≤ 24 h) of tranexamic acid (TXA) was recorded. Our trauma system’s protocols, including prehospital protocols, recommend administering TXA within 3 h of injury for signs of the presence of impending hemorrhagic shock, hypotension (systolic blood pressure ≤ 90 mmHg) and/or clinical suspicion of major hemorrhage. Prehospital TXA dosage was 1 g bolus, in-hospital TXA dosage was also 1 g bolus, and 1 g infusion was repeated over 8 h at discretion of the treating surgeon and/or intensivist.

Denver MOF scores [19] and ARDS Berlin criteria [20] were registered daily up until 28 days or discharge from ICU. Primary outcome was the relation between TXA administration and potentially adverse outcomes such as mortality, thrombo-embolic complications (TEC), MODS, ARDS and infections.

Secondary outcome was potential difference between pre- and in-hospital TXA administration on outcome parameters.

All statistical analyses were performed using IBM SPSS Statistics, version 25.0 (Armonk, NY, USA). Results are presented as median and interquartile range (IQR). Kruskal–Wallis was used to test continuous variables for equality between TXA and patients without TXA, whereas Chi-square or Fisher’s exact test was used to test categorical data. Variables with univariate statistical significance of less than 0.10 were included in a multivariate logistic regression analysis to identify independent risk factors for TXA administration and mortality and presented as odds ratios and 95% confidence intervals. Statistical significance was set at *p* < 0.05.

## Results

In this study, 422 patients (71% male) with a median age of 46 (28–62) years admitted to ICU were included. Ninety-five percent of injuries were caused by a blunt mechanism, 50% was prehospitally intubated, and median ISS was 29 (22–36) with most severe injuries located in the brain (AIS head 3 (1–4)) and chest (AIS chest 3 (2–4)). One hundred and three patients (24%) underwent an urgent laparotomy. Physiology, resuscitation and outcome data are presented in Table 1. In this cohort, 79 (19%) patients died; 57 (72%) of them died of traumatic brain injury (TBI), 7 (9%) died of respiratory insufficiency, 4 (5%) due to exsanguination, 3 (4%) due to cardiac origin, 2 (3%) due to MODS, 2 (3%) due to sepsis, 1 (1%) due to ARDS and 3 (4%) due to miscellaneous causes.

**Table 1:** Demographics, physiology and outcome

	Total population (*n* = 422)	TXA (*n* = 280)	No TXA (*n* = 142)	*p*-Value
Age (years)	46 (28–62)	41 (26–59)	51 (32–67)	0.005*
Male gender	298 (71)	202 (72)	96 (68)	0.37
Blunt MOI	402 (95)	263 (94)	139 (98)	0.09
Prehospital intubation	211 (50)	151(54)	60 (42)	0.002*
Urgent laparotomy	103 (24)	88 (32)	15 (11)	< 0.001*
ISS	29 (22–36)	29 (23–38)	29 (21–34)	0.003*
AIS head	3 (1–4)	3 (0–4)	3 (2–4)	0.22
AIS face	0 (0–2)	0 (0–1)	0 (0–2)	0.28
AIS chest	3 (2–4)	3 (2–4)	3 (2–3)	0.27
AIS abdomen	2 (0–3)	2(0–3)	0 (0–2)	0.004*
AIS pelvis/extremities	2 (0–3)	2 (1–3)	2 (0–3)	0.005*
AIS external	0 (0–1)	0 (0–1)	0 (0–1)	0.31
SBP_ED (mmHg)	120 (98–140)	117 (91–135)	130 (105–144)	< 0.001*
SBP ≤ 90 mmHg_ED	86 (20)	68 (24)	18 (13)	0.005*
Hb_ED (mmol/L)	8.0 (7.2–8.9)	7.8 (7.0–8.9)	8.4 (7.8–9.1)	< 0.001*
pH_ED	7.31 (7.25–7.36)	7.30 (7.23–7.36)	7.33 (7.28–7.39)	< 0.001*
PaC02_ED (mmHg)	46 (41–53)	47 (42–54)	45 (41–51)	0.06
BD _ED (mmol/L)	3.0 (0.0–6.0)	4.0 (1.0–8.0)	2.0 (0.5–5.0)	< 0.001*
PT_ED (sec)	14.6 (13.1–16.9)	14.8 (13.4–17.4)	14.4 (12.7–16.1)	0.04*
*Resuscitation parameters*				
Crystalloids ≤ 8 h (L)	4.5 (2.3–6.2)	5.1 (3.0–7.0)	2.9 (1.4–5.0)	< 0.001*
PRBC ≤ 8 h (U)	1 (0–4)	2 (0–6)	0 (0–0)	< 0.001*
FFP ≤ 8 h (U)	0 (0–4)	2 (0–6)	0 (0–0)	< 0.001*
PLT ≤ 8 h (U)^#^	0 (0–1)	0 (0–1)	0 (0–0)	< 0.001*
Crystalloids ≤ 24 h (L)	7.3 (4.8–10.1)	8.2 (6.0–11.0)	5.5 (3.7–7.9)	< 0.001*
PRBC ≤ 24 h (U)	1 (0–5)	3 (0–7)	0 (0–1)	< 0.001*
PRBC ≥ 10 units ≤ 24 h	44 (10)	42 (15)	2 (1)	< 0.001*
FFP ≤ 24 h (U)	0 (0–5)	2 (0–7)	0 (0–0)	< 0.001*
PLT ≤ 24 h (U)^#^	0 (0–1)	0 (0–1)	0 (0–0)	< 0.001*
*Outcome parameters*				
Ventilator days	6 (2–11)	5 (2–11)	6 (2–11)	0.68
ICU LOS (days)	7 (3–13)	7 (3–13)	7 (3–13)	0.86
H-LOS (days)	20 (11–31)	21 (10–33)	18 (11–29)	0.40
MODS	66 (16)	42 (15)	24 (17)	0.67
ARDS	16 (4)	7 (3)	9 (6)	0.06
Infectious complications	179 (42)	119 (43)	60 (42)	1.0
Thrombo-embolic complications	32 (8)	25 (9)	7 (5)	0.18
Mortality	79 (19)	56 (20)	23 (16)	0.36

Data are expressed in median (IQR) or absolute numbers (%)

*MOI* Mechanism of Injury, *ISS* injury severity score, *AIS* abbreviated injury scale, ED emergency department, *SBP* systolic blood pressure, *Hb* hemoglobin, *PaC02* partial pressure of carbon dioxide in arterial blood, *BD* base deficit, *PT* prothrombin time, *PRBC* packed red blood cells, *ICU* intensive care unit, *LOS* length of stay, *H-LOS* hospital length of stay, *MODS *multiple organ dysfunction syndrome, *ARDS* adult respiratory distress syndrome

* Statistically significant

^#^1 unit of platelets contains five donors

Sixty-six percent of patients received TXA at any point in time. During the 7-year study period, prehospital TXA administration increased (*p* = 0.005, Figure S1A), whereas in-hospital TXA administration did not change over time (*p* = 0.14, Figure S1B). Patients who received TXA were younger, more severely injured, with lower SBP and hemoglobin (Hb) in ED. Further, they were more acidotic and coagulopathic, underwent more often an urgent laparotomy and received more crystalloids and blood products both ≤ 8 and ≤ 24 h than patients who did not receive TXA. There was, however, no difference in outcome between TXA and no-TXA patients (Table 1). There was also no difference in outcome between patients with SBP ≤ 90 mmHg who received TXA and those who did not.

### Subanalysis of patients who received prehospital TXA compared to patients who did not

**Fig. 1:**
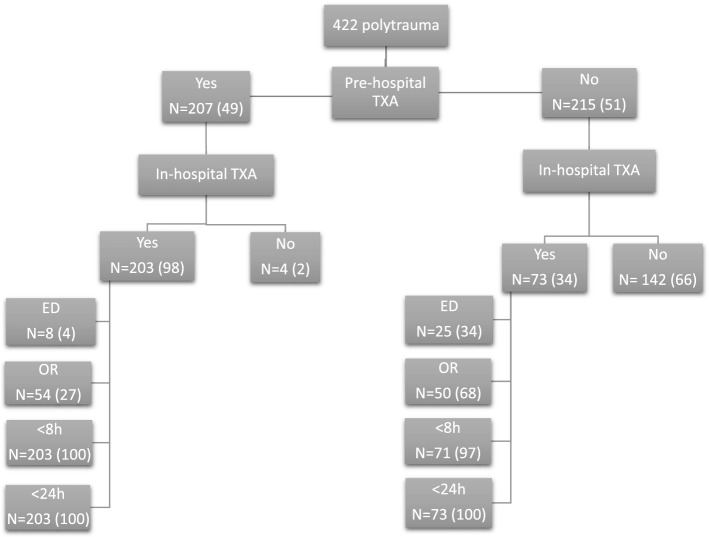
Diagram of polytrauma patients and location of administered tranexamic acid. Data are expressed as absolute numbers (%). *ED* emergency department, *OR* operating room, *TXA* tranexamic acid

Forty-nine percent (*n* = 207) of patients received prehospital TXA, whereas 51% (*n* = 215) did not (Fig. 1). Median time from call to dispatch to ED (prehospital time) was 1:00 h (0:55–1:08), so all prehospital TXA was administered within 1 h after injury. Patients who received prehospital TXA were younger, slightly more severely injured and more often prehospitally intubated. Further, prehospital TXA patients had lower Hb and were more acidotic in ED with higher PaCO2. They received more crystalloids and PRBC ≤ 8 and ≤ 24 h. There was no difference in outcome (Table 2). All four patients (50% had SBP ≤ 90 mmHg) who later died of hemorrhage did not receive prehospital TXA. However, they all did receive TXA in OR.

**Table 2:** Comparison of patients who received prehospital tranexamic acid (TXA) and patients who did not

Demographics *N* = 422	Prehospital TXA (*n* = 207)	No prehospital TXA (*n* = 215)	*p*-Value
Age (years)	40 (26–57)	49 (31–66)	0.004*
Male gender	152 (73)	146 (68)	0.24
Blunt MOI	195 (94)	207 (96)	0.36
Prehospital intubation	134 (65)	77 (36)	< 0.001
Urgent laparotomy	52 (25)	51 (24)	0.82
ISS	29 (22–38)	29 (22–35)	0.01*
AIS head	3 (1–4)	3 (1–4)	0.68
AIS face	0 (0–2)	0 (0–1)	0.43
AIS chest	3 (2–4)	3 (2–4)	0.26
AIS abdomen	2(0–3)	0 (0–3)	0.76
AIS pelvis/extremities	2 (1–3)	2 (0–3)	0.17
AIS external	0 (0–1)	0 (0–1)	0.84
SBP_ED (mmHg)	120 (95–136)	120 (100–140)	0.22
SBP ≤ 90 mmHg_ED	45(22)	41 (19)	0.55
Hb_ED (mmol/L)	7.8 (7.0–8.9)	8.2 (7.4–9.1)	0.008*
pH_ED	7.29 (7.23–7.35)	7.32 (7.27–7.38)	0.001*
PaCO2_ED (mmHg)	48 (42–54)	45 (34–51)	< 0.001*
BD _ED (mmol/L)	3.0 (1.0–7.5)	3.0 (0.0–6.0)	0.22
PT_ED (sec)	14.7 (13.1–17.3)	14.5 (13.2–16.8)	0.76
*Resuscitation parameters*			
Crystalloids ≤ 8 h (L)	4.9 (2.7–6.8)	3.8 (1.9–5.9)	0.001*
PRBC ≤ 8 h (U)	2 (0–5)	0 (0–4)	0.003*
FFP ≤ 8 h (U)	2 (0–5)	0 (0–0)	0.14
PLT ≤ 8 h (U)^#^	0 (0–4)	0 (0–4)	0.28
Crystalloids ≤ 24 h (L)	7.8 (5.6–10.5)	6.7 (4.4–9.5)	0.002*
PRBC ≤ 24 h (U)	2 (0–6)	0 (0–4)	0.002*
PRBC ≥ 10 units ≤ 24 h	28 (14)	16 (8)	0.06
FFP ≤ 24 h (U)	2 (0–6)	0 (0–4)	0.17
PLT ≤ 24 h (U)^#^	0 (0–1)	0 (0–1)	0.21
*Outcome parameters*			
Ventilator days	5 (2–10)	6 (2–11)	0.40
ICU LOS (days)	7 (3–12)	7 (3–14)	0.29
H-LOS (days)	20 (10–31)	20 (11–32)	0.87
MODS	28 (14)	38 (18)	0.28
ARDS	4 (2)	12 (6)	0.07
Infectious complications	85 (41)	94 (44)	0.56
Thrombo-embolic complications	12 (6)	20 (9)	0.20
Mortality	39 (19)	40 (19)	1.0

Data are expressed in median (IQR) or absolute numbers (%)

*MOI* Mechanism of injury, *ISS* injury severity score, *AIS* abbreviated injury scale, *ED* emergency department, *SBP* systolic blood pressure, *Hb* hemoglobin, *PaC02* partial pressure of carbon dioxide in arterial blood, *BD* base deficit, *PT* prothrombin time, *PRBC* packed red blood cells, *ICU* intensive care unit, *LOS* length of stay, *H-LOS* hospital length of stay, *MODS* multiple organ dysfunction syndrome, *ARDS* adult respiratory distress syndrome

*Statistically significant

^#^1 unit of platelets contains five donors

### Subanalysis of patients who received in-hospital TXA compared to patients who did not

Two hundred and seventy-six (65%) patients received in-hospital TXA, whereas 146 did not (Fig. 1). Seventy-four percent had already received prehospital TXA. Patients who received in-hospital TXA were younger, more severely injured with lower SBP and Hb, more acidotic and coagulopathic in ED than patients who did not receive in-hospital TXA. Further, they underwent more often urgent laparotomies and received more crystalloids and blood products ≤ 8 and ≤ 24 h. Again, there was no difference in outcome (Table 3).

**Table 3:** Comparison of patients who received in-hospital TXA and patients who did not

Demographics *N* = 422	In-hospital TXA (*n* = 276)	No in-hospital TXA (*n* = 146)	*p*-Value
Age (years)	42 (26–59)	51 (32–67)	0.01*
Male gender	200 (72)	98 (67)	0.26
Blunt MOI	259 (94)	143 (98)	0.09
Prehospital intubation	149 (54)	62 (43)	0.001*
Urgent laparotomy	88 (32)	15 (10)	< 0.001*
ISS	29 (22–38)	29 (22–34)	0.005*
AIS head	3 (0–4)	3 (2–4)	0.02*
AIS face	0 (0–1)	0 (0–2)	0.58
AIS chest	3 (2–4)	3 (2–3)	0.82
AIS abdomen	2 (0–3)	0 (0–2)	< 0.001*
AIS pelvis/extremities	2 (1–3)	2 (0–3)	< 0.001*
AIS external	0 (0–1)	0 (0–1)	0.96
SBP_ED (mmHg)	117 (91–135)	130 (105–144)	< 0.001*
SBP ≤ 90 mmHg_ED	68(25)	18 (12)	0.002*
Hb_ED (mmol/L)	7.8 (7.0–8.9)	8.4 (7.8–9.1)	< 0.001*
pH_ED	7.30 (7.23–7.36)	7.33 (7.28–7.39)	< 0.001*
PaCO2 (mmHg)	47 (41–54)	45 (41–51)	0.12
BD _ED (mmol/L)	4.0 (1.0–8.0)	2.0 (1.0–5.0)	< 0.001*
PT_ED (sec)	14.9 (13.3–17.5)	14.3 (12.7–16.0)	0.02*
*Resuscitation parameters*			
Crystalloids ≤ 8 h (L)	5.2 (3.1–7.1)	2.9 (1.4–5.0)	< 0.001*
PRBC ≤ 8 h (U)	2 (0–7)	0 (0–0)	< 0.001*
FFP ≤ 8 h (U)	2 (0–6)	0 (0–0)	< 0.001*
PLT ≤ 8 h (U)^#^	0 (0–1)	0 (0–0)	< 0.001*
Crystalloids ≤ 24 h (L)	8.2 (6.1–11.0)	5.5 (3.7–7.9)	< 0.001*
PRBC ≤ 24 h (U)	3 (0–7)	0 (0–1)	< 0.001*
PRBC≥ 10units ≤ 24 h	42 (15)	2 (1)	< 0.001*
FFP ≤ 24 h (U)	2 (0–7)	0 (0–0)	< 0.001*
PLT ≤ 24 h (U)^#^	0 (0–1)	0 (0–0)	< 0.001*
*Outcome parameters*			
Ventilator days	5 (2–11)	6 (2–10)	0.79
ICU LOS (days)	7 (3–13)	7 (3–13)	0.81
H-LOS (days)	21 (10–33)	18 (11–29)	0.54
MODS	42 (15)	24 (16)	0.78
ARDS	7 (3)	9 (6)	0.10
Infectious complications	117 (42)	62 (42)	1.0
Thrombo-embolic complications	25 (9)	7 (5)	0.13
Mortality	55 (20)	24 (16)	0.43

Data are expressed in median (IQR) or absolute numbers (%)

*MOI* Mechanism of injury, *ISS* injury severity score, *AIS* abbreviated injury scale, *ED* emergency department, *SBP* systolic blood pressure, *Hb* hemoglobin, *PaC02* partial pressure of carbon dioxide in arterial blood, *BD* base deficit, *PT* prothrombin time, *PRBC* packed red blood cells, *ICU* intensive care unit, *LOS* length of stay, *H-LOS* hospital length of stay, *MODS* multiple organ dysfunction syndrome, *ARDS* adult respiratory distress syndrome

*Statistically significant

^#^1 unit of platelets contains five donors

**Fig. 2:**
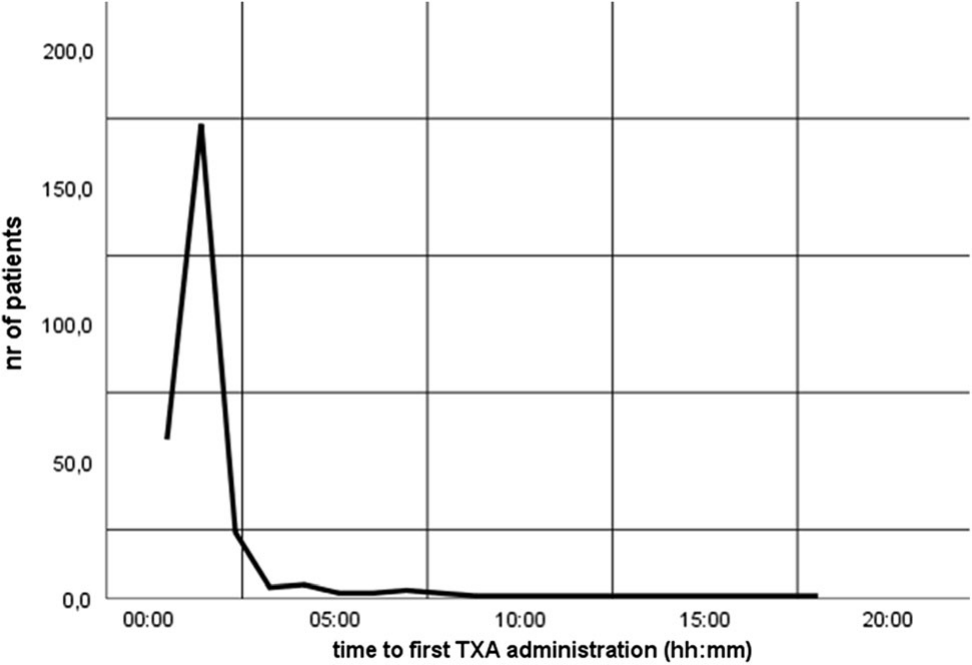
Time to first tranexamic acid (TXA) administration

Median time to TXA was 1:02 h (0:58–1:20). The time frame within TXA was administered is shown in Fig. 2. Ninety-three percent of patients who had TXA received it early (< 3 h) after injury. There was no difference in outcome in patients who had early TXA compared to late TXA (≥ 3 h) (Table S1).

There was no significant difference in TXA dosage in TXA patients who received early TXA compared to late TXA (1 g (1–2) vs. 1 g (1–1), *p* = 0.16). There was also no difference in TXA dosage between patients who developed TEC and those who did not (1 g (1–2) vs. 1 g (1–1), respectively, *p* = 0.20).

Fifty-two percent (45/86) of patients who received prehospital TXA had SBP ≤ 90 mmHg compared to 79% (68/86) of patients who received in-hospital TXA (Table 4). Seventy-eight percent (162/207) of patients received prehospital TXA despite SBP > 90 mmHg in ED, and 75% (208/276) of patients received in-hospital TXA despite SBP > 90 mmHg in ED. Further, 32% (23/73) of patients who had in-hospital TXA had unjustified not received prehospital TXA, and 13% (18/142) of patients who had SBP ≤ 90 mmHg in ED received no TXA at all. Based on systolic blood pressure alone, 22% (45/207) of prehospital TXA and 25% (68/276) of in-hospital TXA were justified (Table 4).

**Table 4:** Relation between systolic blood pressure (SBP) ≤ 90 mmHg in ED and TXA administration

	Prehospital TXA	No prehospital TXA	In-hospital TXA	No in-hospital TXA	Total no. of patients
SBP_ED ≤ 90 mmHg	45	41	68	18	86
SBP_ED > 90 mmHg	162	174	208	128	336
Total no. of patients	207	215	276	146	422

*SBP* systolic blood pressure, *ED* emergency department, *TXA* tranexamic acid

In multivariate analysis, age, hemoglobin, PaCO2 and pH in ED were independent predictors for TXA administration. Age, ISS and base deficit in ED were independent predictors for mortality. TXA, however, was not related to death (Tables 5, 6).

**Table 5:** Independent predictors for TXA administration

Variables in the equation	Β Coefficient	*p*-Value	Odds ratio	95% C.I	
				Lower	Upper
Age	− 0.019	0.001	0.981	0.969	0.992
ISS	0.011	0.306	1.011	0.990	1.033
SBP_ED	− 0.001	0.893	0.999	0.991	1.008
Hb_ED	− 0.041	0.000	0.960	0.939	0.982
BD_ED	0.017	0.099	1.017	0.997	1.037
PT_ED	− 0.001	0.573	0.999	0.995	1.003
PaCO2_ED	− 0.096	0.034	0.908	0.831	0.993
pH_ED	− 0.159	0.023	0.853	0.744	0.978
Constant	125.723	0.019	3.989E+54

**Table 6:** Independent predictors for mortality

Variables in the Equation	Β Coefficient	P-Value	Odds Ratio	95% C.I	
				Lower	Upper
Age	0.046	0.000	1.047	1.030	1.065
Laparotomy	− 0.156	0.695	0.856	0.393	1.865
ISS	0.050	0.000	1.052	1.025	1.079
SBP_ED	0.008	0.095	1.008	0.999	1.018
Hb_ED	− 0.006	0.609	0.994	0.971	1.017
BD_ED	− 0.016	0.000	0.984	0.978	0.990
Thrombo-embolic complications	− 0.453	0.560	0.636	0.139	2.914
TXA	−0.096	0.773	0.908	0.472	1.749
Constant	− 6.625	0.000	0.001		

*ISS* Injury severity score, *SBP* systolic blood pressure, *BD* base deficit, *ED* emergency department, *Hb* hemoglobin, *PT* prothrombin time, *PaCO2* partial pressure of carbon dioxide, *TXA* tranexamic acid

## Discussion

In this cohort of polytrauma patients, there was no difference in outcome between patients who received TXA and those who did not, even though TXA patients were more severely injured with more deranged physiology. Subanalysis of prehospital and in-hospital TXA administration also revealed no difference in outcome. Based on systolic blood pressure alone (SBP ≤ 90 mmHg), large numbers of unjustified prehospital and in-hospital TXA administration were found. However, these large numbers could be slightly overestimated since no data on prehospital SBP were collected and the first collected SBP for this study was in ED. It is possible that prehospital SBP could have been higher and decreased during transport. Moreover, since the original inclusion to administer TXA was a rather vague and subjective description of “signs of the presence of impending hemorrhagic shock and/or clinical suspicion of major hemorrhage,” in this study SBP ≤ 90 mmHg was used as most objective measurement of hemorrhagic shock to be able to calculate whether TXA administration was justified. This strict inclusion of SBP ≤ 90 mmHg might label TXA as “unjustified” in some patients with normal SBP even though they were in imminent shock. Especially in prehospital settings, it could be difficult to diagnose early signs of hemorrhagic shock, and a “better safe than sorry” attitude is often adopted. Another reason for TXA administration in patients without hypotension might be explained by a previously described phenomenon in which severely injured patients in smaller service areas with short transport times do not have deranged physiologic parameters on arrival in ED. These patients did not have the time to deteriorate, because they were in the hospital before blood pressure, BD and hemoglobin will change distinctly [21, 22].

This liberal approach of prehospital TXA administration even increased over time during the 7-year study and has recently also been described by Kheirbek et al. Despite large numbers of unjustified TXA use, they also did not demonstrate difference in TEC [23].

In addition to the rationale of unjustified TXA administration, it is also intriguing why patients with signs of shock did not receive TXA. A few of these patients had shock based on other causes than hemorrhage such as neurogenic shock based on high spinal cord lesion. Four patients (5%) who died due to hemorrhagic shock did not receive prehospital TXA, nor in ED. They all did receive TXA in OR. It could be debated that time was so critical in these exsanguinating patients that there was simply no time to administer TXA prehospitally nor in ED. This was contradicted by reviewing both prehospital transport times and time from ED to OR in these patients, and there was no difference compared to other patients who needed urgent surgery. It remains unclear whether these patients would have survived if they had received TXA earlier.

Almost all patients who received TXA for the first time had it within the recommended 3 h after injury. There was no difference in dosage between patients who had early TXA and those who had late TXA nor was there any difference in outcome suggesting that both dosage and timing of TXA did not influence outcome.

Our data are in line with several other reports in the literature suggesting that TXA in a polytrauma population was not associated with increased TEC and mortality even if it was administered liberally [10–13]. However, current data are in contrast to other studies that demonstrated increased mortality after TXA [6–9]. A possible explanation for these seemingly contradicting data was proposed by Moore et al. suggesting that outcome after TXA might be related to fibrinolytic state of the patient with least expected benefit from TXA in patients with physiological fibrinolysis [9, 24]. In our hospital, viscoelastic tests are not routinely used in trauma. However, in a previous study thromboelastography in severely injured patients (who were part of the same cohort used in this study) showed no abnormalities [25]; therefore, it could be assumed that the patients in this study had physiological levels of fibrinolysis. Nevertheless, there was no difference in mortality between patients who received TXA and patients who did not in this study.

Despite being more severely injured TXA patients had no difference in outcome suggesting that TXA has ameliorated outcome. This should be concluded with caution; First of all, even if only patients with SBP ≤ 90 mmHg in ED were analyzed, there was no difference in morbidity and mortality between patients with and without TXA. Further, the numbers of exsanguinating patients were very low since only four patients died of hemorrhage. Additionally, it remains to be seen whether TXA is truly advantageous in settings with small service areas with short transport times and with the immediate availability of blood products and operating room to control hemorrhagic shock.

In this cohort of polytrauma patients, many patients also sustained TBI (AIShead 3 (1–4)). TBI was also the main cause of death in this population (72%). Data even suggest that prehospital TXA was often given in patients with TBI since patients who received prehospital TXA had higher AIShead, were more often prehospitally intubated and had higher PaCO2 with similar pH in ED (suggestive for prehospital hypoventilation) than patients who did not receive prehospital TXA. Several studies have reported various effects of TXA on outcome in TBI from depending on brain injury severity and timing of TXA administration [26] to no difference in outcome after prehospital TXA [27], or even a potential harmful effect of prehospital TXA on mortality in severe TBI patients [28]. The effect of TXA on TBI in this polytrauma population was beyond the scope of this paper. Future research will focus on the effect of TXA on TBI.

A few limitations need to be acknowledged: First of all, this was a single-institution study. Further, clinicians who were treating these patients were also the researchers. Another limitation is that no details on comorbidities nor any data on prehospital and in-hospital Glasgow Coma Scale were collected.

In conclusion, TXA patients had similar outcome compared to patients without TXA despite being more severely injured. There was a liberal use of both prehospital and in-hospital TXA with large numbers of patients receiving TXA without hypotension. Hemorrhagic shock was indication for prehospital TXA in only half the patients, whereas in-hospital TXA was given based on suspicion of hemorrhagic shock in the vast majority of patients. Caution should be exercised since it can be difficult to select the right patient for TXA especially in severely injured patients who do not show grossly signs of deranged physiology prehospitally or on arrival in ED yet.

## Supplementary Information

Below is the link to the electronic supplementary material.

Supplementary file1 (DOCX 40 kb)

Supplementary file2 (DOCX 19 kb)

## Data Availability

The datasets supporting the conclusions of this article are available upon reasonable request from the corresponding author.
