# Supplementary figures and images for: Does Liberal Prehospital and In-Hospital Tranexamic Acid Influence Outcome in Severely Injured Patients? A Prospective Cohort Study
Source: World J Surg. 2021 Apr 29;45(8):2398–407. doi: 10.1007/s00268-021-06143-y (PMC8083099; doi:10.1007/s00268-021-06143-y)

**Figure S1.** Prehospital (A) and in-hospital (B) tranexamic acid (TXA) administration over time.

A.


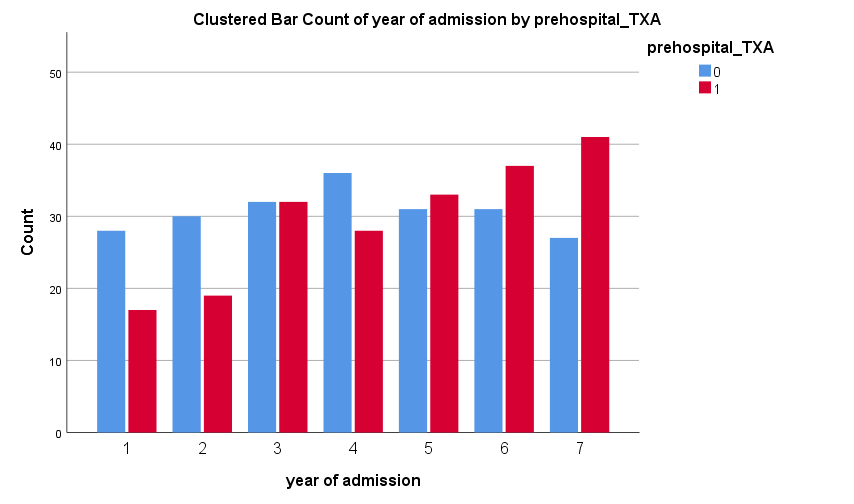
 P=0.005

B.


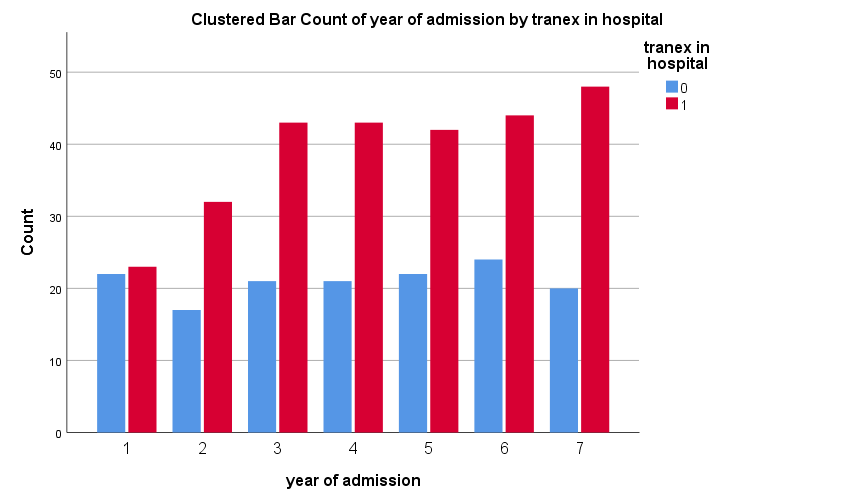
P=0.14

Supplement: Supplementary file 1 — Supplementary file1 (DOCX 40 kb) [file 268_2021_6143_MOESM1_ESM.docx]
