# Supplementary material for: Does Liberal Prehospital and In-Hospital Tranexamic Acid Influence Outcome in Severely Injured Patients? A Prospective Cohort Study
Source: World J Surg. 2021 Apr 29;45(8):2398–407. doi: 10.1007/s00268-021-06143-y (PMC8083099; doi:10.1007/s00268-021-06143-y)
Supplement: Supplementary file 2 — Supplementary file2 (DOCX 19 kb) [file 268_2021_6143_MOESM2_ESM.docx]

**Table S1.** Demographics, physiology and outcome in early (<3h) vs. late (>3h) TXA administration

|  | TXA<3h  (n=261) | TXA>3h  (n=19) | P-value |
| --- | --- | --- | --- |
| Age (years) | 40 (26-58) | 57 (38-73) | 0.02* |
| Male gender | 188 (72) | 14 (74) | 1.0 |
| Blunt MOI | 245 (94) | 18 (95) | 1.0 |
| Prehospital intubation | 148 (57) | 3 (16) | 0.002* |
| Urgent laparotomy | 82 (31) | 6 (32) | 1.0 |
| ISS | 29 (22-38) | 29 (25-38) | 0.80 |
| AIS head | 3 (0-4) | 3 (0-4) | 0.89 |
| AIS face | 0 (0-1) | 0 (0-1) | 0.72 |
| AIS chest | 3 (2-4) | 3 (1-4) | 0.67 |
| AIS abdomen | 2(0-3) | 1 (0-2) | 0.24 |
| AIS pelvis/extremities | 2 (2-3) | 2 (0-3) | 0.12 |
| AIS external | 0 (0-1) | 0 (0-1) | 0.65 |
| SBP_ED (mmHg) | 118 (91-134) | 110 (90-136) | 0.55 |
| SBP<90 mmHg_ED | 63 (24) | 5 (26) | 0.79 |
| Hb­­_ED (mmol/L) | 7.8 (7.0-8.9) | 7.8 (6.8-9.3) | 0.71 |
| pH_ED | 7.30 (7.22-7.35) | 7.34 (7.28-7.40) | 0.04* |
| PaC02_ED (mmHg) | 48 (42-54) | 44 (36-51) | 0.07 |
| BD _ED (mmol/L) | -4.0 (-8.0--1.0) | -3.0 (-5.0-0.0) | 0.20 |
| PT_ED (sec) | 15.0 (13.3-17.6) | 14.2 (12.9-15.6) | 0.06 |
| Resuscitation parameters |  |  |  |
| Crystalloids<8h (L) | 5.1 (2.8-6.9) | 5.5 (4.3-9.9) | 0.58 |
| PRBC<8h (U) | 2 (0-6) | 3 (0-7) | 0.98 |
| FFP<8h (U) | 2 (0-6) | 3 (0-4) | 0.90 |
| PLT<8h (U)^#^ | 0 (0-1) | 0 (0-1) | 0.95 |
| Crystalloids <24h (L) | 8.1 (5.7-10.7) | 9.3 (7.1-13.1) | 0.08 |
| PRBC<24h (U) | 3 (0-7) | 5 (0-9) | 0.45 |
| PRBC >10 units <24h | 40 (15) | 2 (11) | 0.75 |
| FFP< 24h (U) | 2 (0-7) | 3 (0-6) | 0.71 |
| PLT< 24h (U)^#^ | 0 (0-1) | 0 (0-2) | 0.65 |
| Outcome parameters |  |  |  |
| Ventilator days | 5 (2-10) | 7 (2-11) | 0.67 |
| ICU LOS (days) | 7 (3-13) | 11 (4-19) | 0.07 |
| H-LOS (days) | 20 (10-31) | 30 (15-39) | 0.14 |
| MODS | 38 (15) | 4 (21) | 0.50 |
| ARDS | 5 (2) | 2 (11) | 0.08 |
| Infectious complications | 111 (43) | 8 (42) | 1.0 |
| Thrombo-embolic complications | 23 (9) | 2 (11) | 0.68 |
| Mortality | 54 (21) | 2 (11) | 0.38 |

Data are expressed in median (IQR) or absolute numbers (%), *=statistically significant

^#^ 1 unit of platelets contains 5 donors

MOI=Mechanism of Injury, ISS=Injury Severity Score, AIS=Abbreviated Injury Scale, ED=Emergency Department, SBP=systolic blood pressure, Hb=hemoglobin, PaC02= partial pressure of carbon dioxide in arterial blood, BD=Base Deficit, PT=prothrombin time, PRBC=packed red blood cells, ICU= Intensive Care Unit, LOS=length of stay, H-LOS=hospital length of stay, MODS=Multiple Organ Dysfunction Syndrome, ARDS=Adult Respiratory Distress Syndrome.
